# Genomic epidemiology and anti-biofilm mechanisms of Lactobacillus in ST11 carbapenem-resistant *Klebsiella pneumoniae* in China

**DOI:** 10.3389/fmicb.2025.1619621

**Published:** 2025-07-02

**Authors:** Shuang Li, Duo Zhang, Xindan Zhang, Xiaohan Ma, Shuai Zheng, Di Zhou, Qinlong Hou, Gen Li, Huiming Han

**Affiliations:** ^1^Department of Science and Education, Affiliated Hospital of Beihua University, Jilin, China; ^2^The School of Basic Medicine, Beihua University, Jilin, China; ^3^The Center for Infection and Immunity, Beihua University, Jilin, China

**Keywords:** ST11 *Klebsiella pneumoniae*, biofilm, Lactobacillus, probiotics, metabolomics, multidrug resistance

## Abstract

**Background:**

The ST11 clone of carbapenem-resistant *Klebsiella pneumoniae* (CRKP) has emerged as a major public health threat, driving hospital outbreaks across China and contributing to chronic infections through robust biofilm formation. The scarcity of effective treatment options poses a critical challenge to clinical management.

**Methods:**

To address this issue, we conducted an integrated genomic epidemiological and metabolomic study of ST11 CRKP isolates collected from 13 hospitals in eastern and central China between 2014 and 2020. A total of 2,805 clinical isolates were screened, and 334 ST11 strains were identified using MALDI-TOF mass spectrometry and whole-genome sequencing. Biofilm formation was assessed through microtiter plate assays, while co-culture experiments with Lactobacillus fermentum and Lactobacillus gasseri were performed to evaluate anti-biofilm activity. Scanning electron microscopy (SEM) and non-targeted metabolomics were used to explore structural and metabolic changes.

**Results:**

Genomic analysis revealed alarming resistance rates exceeding 90% to *β*-lactams, fluoroquinolones, and aminoglycosides among ST11 isolates. Distinct regional distributions of capsular types were observed, with K64 predominant in the east and K47 more common in central China. Biofilm assays showed that 97.6% (326/334) of isolates were biofilm producers. Co-culture with *L. fermentum* and *L. gasseri* significantly reduced biofilm biomass by 41.3–58.7% (*p* < 0.001), and SEM confirmed biofilm structural disruption. Metabolomic analysis revealed that *L. fermentum* disrupted purine biosynthesis and aminoacyl-tRNA metabolism, while *L. gasseri* inhibited folic acid synthesis (FDR = 0.017) and the phosphotransferase system.

**Conclusion:**

This study reveals critical insights into the clonal spread and biofilm-associated metabolic vulnerabilities of ST11 CRKP. The findings highlight the therapeutic potential of Lactobacillus-based interventions and pave the way for novel probiotic-assisted and plasmid-targeted strategies against antimicrobial-resistant bacteria.

## 1 Introduction

Carbapenem-resistant *Klebsiella pneumoniae* (CRKP), particularly the ST11 clone producing carbapenemases, has emerged as a critical global public health threat, driving hospital-acquired infections with its multidrug resistance (MDR) (Jin et al., 2021; Zhou Y. et al., 2023). The ST11 clone, now dominant in China, has rapidly spread worldwide, fueled by the production of carbapenemases (especially KPC-2), extended-spectrum *β*-lactamases (ESBLs), and plasmid-mediated resistance genes, rendering it resistant to nearly all β-lactam antibiotics and severely limiting therapeutic options (Li et al., 2023; Liao et al., 2023; Wang et al., 2023). Studies have revealed distinct regional epidemiological patterns of ST11-CRKP in China, with the K64 capsule type predominating in the eastern region and the K47 type more prevalent in the central region, suggesting region-specific evolutionary trajectories. These findings underscore the need for a deeper understanding of the genomic epidemiology and transmission dynamics of ST11-CRKP.

Biofilm formation further exacerbates the challenges posed by CRKP infections. Biofilms not only enhance bacterial resistance to antibiotics but also facilitate the horizontal transfer of resistance genes (Di Domenico et al., 2020; Guan et al., 2023). ST11-CRKP isolates exhibit biofilm-forming ability, which is closely associated with persistent device-related infections and recurrent bacteremia (Chen C. et al., 2024; Chen T. et al., 2024; Ye et al., 2022). Although novel *β*-lactam/β-lactamase inhibitor combinations, such as ceftazidime-avibactam, show promise, their efficacy against CRKP within biofilms remains limited (Mahmud et al., 2022). This highlights the urgent need for alternative strategies to combat biofilm-mediated persistent infections and resistance to antibiotics.

Probiotic interventions, particularly those involving Lactobacillus species, have gained attention for their potential to disrupt biofilms (Wasfi et al., 2018; Zeng et al., 2022). Previous studies have demonstrated that *Lactobacillus fermentum* and *Lactobacillus gasseri* can inhibit biofilm formation in Enterobacteriaceae by interfering with quorum sensing and extracellular polymeric substance (EPS) production. However, the metabolic pathways underlying the anti-biofilm effects of Lactobacillus on CRKP remain poorly characterized (Kim et al., 2009). Non-targeted metabolomics has emerged as a powerful tool for investigating host–microbe–pathogen interactions, but its application in studying Lactobacillus-CRKP interactions remains in its early stages.

Building on these gaps, this study addresses two critical questions: (1) What are the genomic epidemiological characteristics of ST11-CRKP in eastern and central China, including region-specific resistance gene profiles and plasmid transmission dynamics? (2) What are the metabolic mechanisms by which Lactobacillus mediates biofilm inhibition? To answer these questions, we integrated whole-genome sequencing of 334 ST11 isolates with LC–MS/MS metabolomics to explore the metabolic interactions between CRKP and Lactobacillus. Our findings not only elucidate the molecular drivers of regional CRKP transmission but also reveal strain-specific transmission patterns. Furthermore, we identify probiotic metabolic targets for biofilm control, offering novel and feasible strategies to address antibiotic resistance and biofilm persistence in CRKP infections. This study bridges critical gaps in the field, providing a foundation for the development of innovative therapeutic approaches.

## 2 Materials and methods

### 2.1 Genomic epidemiology of ST11-CRKP

#### 2.1.1 Isolation and identification of strains

A total of 2,805 bacterial samples were collected from 13 hospitals in eastern and central China between 2014 and 2020. All strains were cultured using the streak plate method on Mueller-Hinton agar (MHA) and incubated at 37°C for 18 h. Single colonies with good growth were selected from different colony morphologies and further purified, followed by identification using Matrix-Assisted Laser Desorption/Ionization Time-of-Flight Mass Spectrometry (MALDI-TOF MS).

#### 2.1.2 Antimicrobial susceptibility testing

Antimicrobial susceptibility was assessed using the agar dilution method and broth microdilution method, testing 14 antimicrobial agents against ST11-CRKP. The tested antibiotics included imipenem, meropenem, piperacillin/tazobactam, trimethoprim/sulfamethoxazole, cefotaxime, ceftazidime, cefepime, cefoperazone, amikacin, tigecycline, gentamicin, ciprofloxacin, levofloxacin, and polymyxin B. The results for tigecycline and polymyxin B were interpreted according to the European Committee on Antimicrobial Susceptibility Testing (EUCAST) guidelines (Giske et al., 2022), while the remaining drugs were interpreted based on the 2023 guidelines of the Clinical and Laboratory Standards Institute (Rai et al., 2023). *Escherichia coli* ATCC 25922, *Klebsiella pneumoniae* ATCC 700603, and *Pseudomonas aeruginosa* ATCC 27853 were used as quality control strains (CLSI, 2023).

#### 2.1.3 Whole genome sequencing (WGS)

High-throughput sequencing was performed by Beijing Novogene Technology Co., Ltd. using the Illumina NovaSeq 6,000 platform. Raw data were processed using SPAdes 3.10.1 software, and after removing redundant information, genome sequences were assembled. The whole genome data were uploaded to the Center for Genomic Epidemiology^1^, where ResFinder 4.1 was used to detect antibiotic resistance genes, and PlasmidFinder 2.1 was used to determine plasmid replicon types. Sequence Type (ST) was determined by comparing with the MLST 2.0 database. Virulence genes were identified by aligning genome sequences with the VFDB database^2^. Core genome alignment of different strains was performed using Roary software, and a phylogenetic tree was constructed using MEGA software and visualized using iTOL (Liu et al., 2024).

#### 2.1.4 Biofilm formation assessment

The bacterial strain was streaked onto MHA plates using the quadrant streak method and incubated overnight at 37°C. Then, 5 mL of LB liquid medium was added to each 15 mL centrifuge tube. A single colony was picked and gently ground on the inner wall of the tube, followed by incubation at 37°C in a shaker for 6 h. After centrifugation at 15,000 rpm for 20 min, the supernatant was discarded, and the pellet was retained.

Next, an appropriate number of turbidimetric tubes were prepared and labeled. Each tube was filled with 2 mL of LB liquid medium, and the pellet obtained from step 3 was used to adjust the bacterial suspension to a 0.5 McFarland turbidity. The adjusted bacterial suspension was distributed into a 96-well plate, 200 μL per well. An LB medium negative control group was also set for each bacterium, with all experimental groups incubated at 37°C for 24 h (note: three replicate wells for each bacterium).

After incubation, the liquid was removed from the 96-well plate, and each well was gently washed three times with 150 μL PBS (pH 7.2), followed by air drying. Then, 150–200 μL of methanol was added to each well, and the plate was left to fix for 15 min. After removing the methanol, the plate was air-dried. Next, the wells were stained with 0.1% crystal violet solution at room temperature for 15 min. The wells were then washed three to four times with PBS or deionized water and air-dried again.

Finally, 100 μL of DMSO was added to each well, incubated for 5 min, and gently shaken to dissolve the attached crystal violet. After mixing thoroughly, the absorbance of the sample solution at 595 nm was measured using a microplate reader. The experimental data were recorded and calculated.

Result Interpretation: The average OD value of the negative control group (ODc) was used as a reference. If the OD value is less than ODc, it indicates that the bacteria do not form biofilms. If the OD value is between ODc and 2 × ODc, it indicates weak biofilm formation. If the OD value is between 2 × ODc and 4 × ODc, it indicates moderate biofilm formation. If the OD value is greater than 4 × ODc, it indicates strong biofilm formation. All results are expressed as the mean ± standard deviation.

#### 2.1.5 Scanning electron microscopy (SEM) for biofilm analysis

ST11 strains were inoculated into MHB medium and incubated at 37°C with shaking for 24 h. After centrifugation at 4°C, 5000 × g, bacterial cells were fixed in 2.5% glutaraldehyde (pre-cooled to 4°C) for 2 h. Cells were dehydrated through a 50–100% ethanol gradient and then subjected to critical point drying (Leica EM CPD300) and platinum ion sputtering (Hitachi E-1045). Biofilm three-dimensional structures were observed using a Hitachi TM4000 PLUS scanning electron microscope (15 kV, SE mode).

### 2.2 Exploration of the metabolic mechanisms of *Lactobacillus fermentum* and *Lactobacillus gasseri* in inhibiting biofilm formation

#### 2.2.1 Selection and identification of strains

In this study, 7 strong biofilm-forming ST11-CRKP strains were selected from 334 *Klebsiella pneumoniae* (cKP) isolates: 3 from blood samples and 4 from fecal samples. Additionally, 23 breast milk samples from different patients were collected. These breast milk samples were cultured in MRS broth under anaerobic conditions at 37°C. A total of 50 μL of the enriched bacterial liquid was evenly spread onto MRS agar plates and incubated under anaerobic conditions overnight. Single colonies with different morphologies were selected for further purification and identified using the MALDI-TOF MS method.

#### 2.2.2 Biofilm formation assay with *Lactobacillus* supernatant and ST11-CRKP co-cultures

First, ST11-CRKP strains were streaked onto MHA agar plates using the three-zone streaking method and incubated at 37°C for 18–24 h. Simultaneously, Lactobacillus fermentum and Lactobacillus gasseri were inoculated into MRS liquid medium and statically cultured under anaerobic conditions at 37°C for 18–24 h. Fifteen mL sterile centrifuge tubes were prepared, each containing 5 mL of LB broth medium. A single colony was uniformly ground against the tube wall, vortex-mixed at medium speed for 20 s, and then incubated in a 37°C shaking incubator (220 rpm) for 6 h. The Lactobacillus cultures were centrifuged at 15,000 × g and 4°C for 20 min, and the sterile supernatant was collected and stored.

The ST11-CRKP bacterial suspension was adjusted to 0.5 McFarland standard turbidity (~1.5 × 10^8^ CFU/mL) using LB medium, followed by a 100-fold serial dilution. A 100 μL aliquot of the diluted suspension was added to a 96-well plate, with 100 μL of Lactobacillus supernatant added to each well. Three experimental groups were established: (1) LB broth + CRKP (positive control), (2) MRS broth + CRKP (matrix control), and (3) LB broth (negative control). Each group included three technical replicates and was repeated in three independent experiments. The 96-well plate was statically incubated at 37°C for 24 h. The absorbance at 595 nm (OD_595_) was measured using a microplate reader (SpectraMax M5, Molecular Devices), with triplicate readings per well averaged after vortex mixing.

#### 2.2.3 Scanning electron microscopy (SEM) of *Lactobacillus* supernatant and ST11-CRKP co-culture biofilms

The 0.5 McFarland turbidity ST11-CRKP suspension cultured in LB broth was mixed with the supernatant of *Lactobacillus fermentum* and *Lactobacillus gasseri* cultured in MRS broth at a 1:1 ratio. The mixture was placed in centrifuge tubes and incubated in a 37°C incubator for 24 h. Once you have centrifuged the sample, quickly fix the settled bacteria in 2.5% fresh glutaraldehyde for 2 h. Then rinse the bacteria with distilled water three times, then dehydrate using increasing concentrations of ethanol: 20 min in 50% ethanol, then 20 min in 75% ethanol, followed by 20 min in 85% ethanol, then 20 min in 95% ethanol, and finally, two rounds of 20 min in 100% ethanol. After that, proceed with critical point drying, ion sputtering treatment, and then observe under a microscope.

#### 2.2.4 Untargeted metabolomics analysis

ST11-CRKP was inoculated into LB liquid medium and incubated at 37°C with shaking at 200 rpm until the logarithmic growth phase (OD600 = 0.5–0.7). After incubation, the bacterial liquid was collected by centrifugation at 4000 rpm for 10 min. The bacterial pellet was adjusted to 0.5 McFarland turbidity, resuspended in fresh LB liquid medium, and mixed with *Lactobacillus* supernatant at a 1:1 ratio. The co-culture was incubated at 37°C for 24 h. After the co-culture, the bacterial cells were again collected by centrifugation at 4000 rpm for 10 min, washed three times with sterile PBS (pH = 7.2), snap-frozen in liquid nitrogen, and stored at −80°C. The samples stored at −80°C were thawed in a 4°C ice bath, 300 μL of 80% methanol solution was added, and the samples were snap-frozen in liquid nitrogen for 5 min, then thawed again. The samples were vortexed for 30 s, ultrasonic extracted for 6 min, and then centrifuged at 5000 rpm for 1 min at 4°C. The supernatant was collected and lyophilized to a dry powder, which was then re-dissolved in a 10% methanol solution for liquid chromatography-mass spectrometry (LC–MS) analysis.

To ensure data quality, equal amounts of sample from each group were mixed to prepare quality control (QC) samples. Hypersil Gold C18 column (Thermo Fisher Scientific, 100 × 2.1 mm, 1.9 μm) was used, with the column temperature set at 40°C and a flow rate of 0.2 mL/min. In positive ion mode, the mobile phase A was 0.1% formic acid aqueous solution, and phase B was methanol; in negative ion mode, mobile phase A was 5 mM ammonium acetate solution (pH = 9.0), and phase B was methanol. The gradient elution program was as follows: initial A: B = 98:2, maintained for 1.5 min; after 3 min, A decreased to 15% and B increased to 85%; after 10 min, A decreased to 0% and B increased to 100%; at 10.1 min, the initial ratio A: B = 98:2 was restored and maintained for 1.9 min. Mass spectrometry was performed by alternating scans in positive and negative ion modes over the m/z range of 100–1,500, with electrospray ionization (ESI) at a spray voltage of 3.5 kV.

The raw data were processed using Linux system (CentOS 6.6), R, and Python for peak extraction, alignment, and normalization. Metabolite annotation was performed using the KEGG, HMDB, and LIPIDMaps databases. Multivariate statistical analysis was performed using metaX software, including Principal Component Analysis (PCA) and Partial Least Squares Discriminant Analysis (PLS-DA), and calculation of Variable Importance Projection (VIP) values. Univariate analysis was performed using the t-test to assess significance, and fold change (FC) was calculated, with the selection criteria of VIP > 1, *p* < 0.05, and FC ≥ 2 or ≤ 0.5. Differential metabolites were visualized using volcano plots created by ggplot2, and metabolic pathway enrichment analysis was performed using the KEGG database (*p* < 0.05). Correlation analysis was performed using R, and the enrichment results were displayed as bubble plots.

## 3 Results

### 3.1 Strain selection, identification, and antibiotic susceptibility results

**Figure 1:**
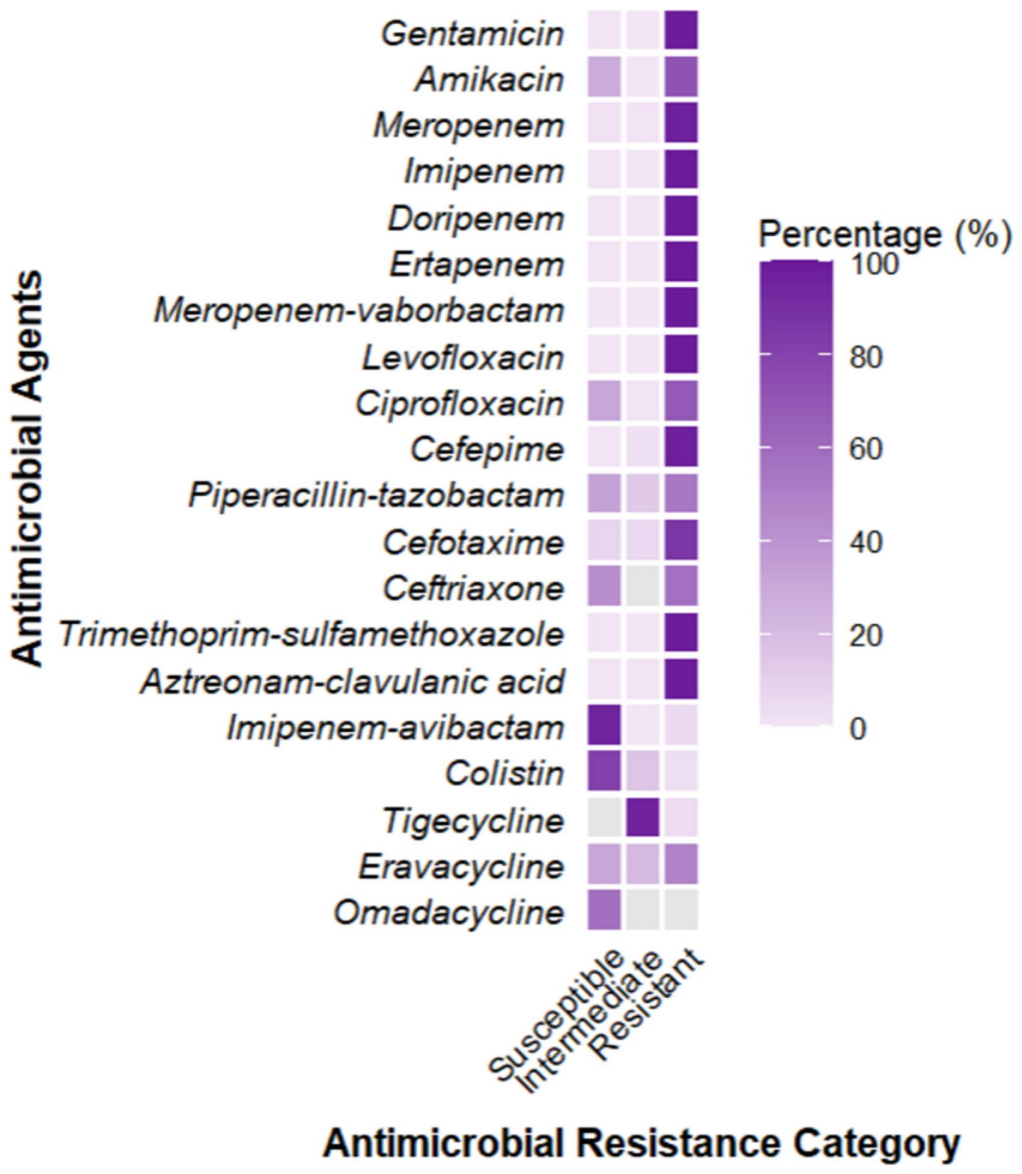
Results of the antimicrobial susceptibility test for the microbial strain.

**Figure 2:**
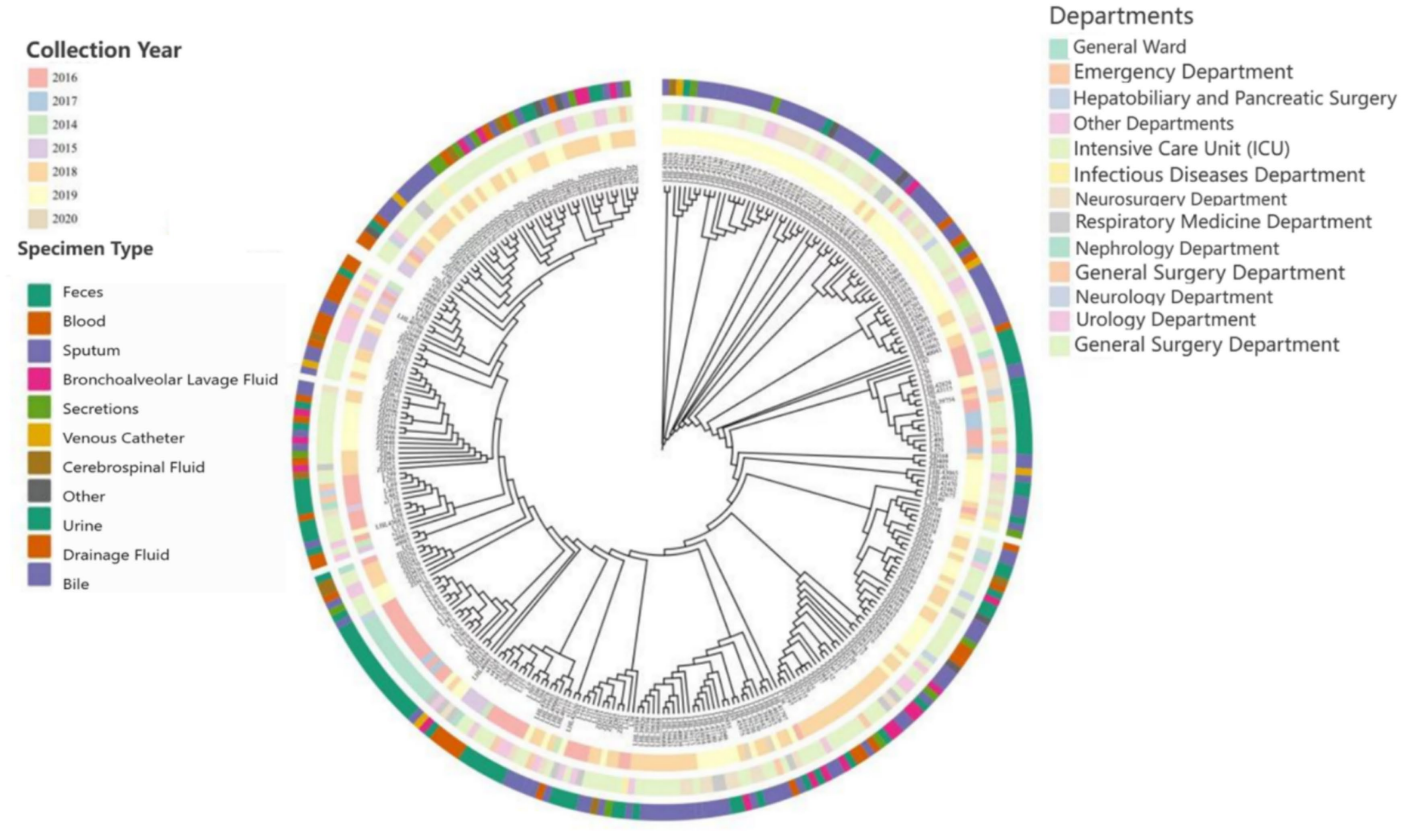
Diagram showing the phylogenetic relationships of ST11-cKP.

A total of 2,805 samples were collected from 13 hospitals in Eastern and Central China between 2014 and 2020. After culturing on MHA using the streak plate method, single colonies were selected for mass spectrometry identification. A total of 669 KP strains were screened, from which 425 cKP strains were further selected using PCR. Finally, based on next-generation sequencing results, 334 ST11-cKP strains were chosen for further experiments. The antibiotic susceptibility testing results for ST11-cKP are shown in Figure 1. We generated a phylogenetic tree for the collected ST11 type cKP to look at the phylogenetic relationships between different regions and hospitals. You can see the results in Figure 2. We found that strains from the same area were more genetically similar.

Based on the *in vitro* antibiotic susceptibility interpretation criteria (1–8), the 334 strains showed high resistance to multiple antibiotics. Over 97% of the strains were resistant to aztreonam, imipenem, meropenem, ceftriaxone, cefotaxime, ceftazidime, cefepime, levofloxacin, ciprofloxacin, piperacillin-tazobactam, amoxicillin-clavulanic acid, and cefoperazone. Among these, the resistance rates to ceftazidime-avibactam, tigecycline, and polymyxin B were relatively lower. Regional analysis showed that the resistance rates of cKP strains in Central China were generally higher than those in Eastern China (Table 1).

**Table 1:** Analysis of how bacterial strains from the Central Region and East China respond to antibiotics.

Drug Name	Eastern China			Central China		
	Susceptible	Intermediate	Resistant	Susceptible	Intermediate	Resistant
Gentamicin	2	1	195	0	0	136
Amikacin	1	0	197	0	1	135
Meropenem	5	0	193	0	3	133
Imipenem	2	0	196	0	0	136
Doripenem	2	0	196	0	0	136
Ertapenem	2	0	196	0	0	136
Meropenem-vaborbactam	2	1	195	0	1	135
Levofloxacin	0	0	198	0	0	136
Ciprofloxacin	1	0	197	0	0	136
Amikacin	97	1	100	5	1	130
Cefepime	91	0	107	3	0	133
Piperacillin-tazobactam	0	8	190	0	0	136
Cefotaxime	68	38	92	42	6	88
Ceftriaxone	4	12	182	32	7	97
Trimethoprim-sulfamethoxazole	57	-	141	25	-	111
Aztreonam-clavulanic acid	3	0	195	0	0	136
Imipenem-avibactam	195	-	5	121	-	16
Colistin	-	191	7	-	129	7
Tigecycline	152	43	3	114	9	3
Eravacycline	128	-	-	84	-	-
Omadacycline	45	37	116	57	33	46

### 3.2 Antibiotic resistance genes carried by carbapenemase-producing *Klebsiella pneumoniae* strains

Next,-generation sequencing combined with the ResFinder tool was used for the identification of resistance genes, and the results were highly consistent with PCR detection data. A total of 334 strains were analyzed, including 198 from Eastern China and 136 from Central China.

In Eastern China, 197 strains carried the *bla*_KPC-2_ gene, 2 strains carried both *bla*_KPC_-2 and *bla*_NDM-1_, and 1 strain carried only *bla*_NDM-1_. In Central China, all strains carried the *bla*_KPC-2_ gene, with 1 strain carrying both *bla*_KPC-2_ and *bla*_NDM-1_, and another strain carrying both *bla*_KPC-2_ and *bla*_NDM-5_. Additionally, no other types of carbapenemase resistance genes were detected in either region.

All cKP strains carried broad-spectrum *β*-lactamase (ESBLs)-related resistance genes, and most strains carried at least two types of ESBL-encoding genes. The most common ESBL genes in both Eastern and Central China were *bla*_SHV_. In Eastern China, the predominant gene was *bla*_SHV-182_, followed by *bla*_SHV-11_, *bla_SHV-178_*, and *bla*_*SHV-160*._

In Central China, the predominant gene was *bla_SHV-178_*, followed by *bla*_SHV-11_, *bla*_SHV-12_, and *bla*_SHV-155_ (Table 2).

**Table 2:** Genes that confer resistance associated with ESBL.

Drug resistance genes	Eastern China		Central China	
	Number of positive strains	Percentage (%)	Number of positive strains	Percentage (%)
*bla*_CTX-M_	109	55.05	129	94.85
*bla*_CTX-M-3_	8	4.04	4	2.94
*bla*_CTX-M-14_	9	4.55	1	0.74
*bla*_CTX-M-147_	3	1.52	0	0
*bla*_CTX-M-65_	93	46.97	125	91.91
*bla*_CTX-M-105_	2	1.01	0	0
*bla*_CTX-M-99_	3	1.52	0	0
*bla*_CTX-M-134_	2	1.01	0	0
*bla*_CTX-M-83_	1	0.51	0	0
*bla*_CTX-M-15_	0	0	18	13.24
*bla*_CTX-M-80_	0	0	1	0.74
*bla*_CTX-M-66_	0	0	1	0.74
*bla*_SHV_	191	96.46	130	95.59
*bla*_SHV-11_	87	43.94	40	29.41
*bla*_SHV-12_	4	2.02	23	16.91
*bla*_SHV-172_	22	11.11	8	5.88
*bla*_SHV-160_	26	13.13	5	3.68
*bla*_SHV-159_	1	0.51	0	0
*bla*_SHV-182_	98	49.49	0	0
*bla*_SHV-178_	29	14.65	89	65.44
*bla*_SHV-153_	0	0	1	0.74
*bla*_SHV-129_	2	1.01	3	2.21
*bla*_SHV-121_	0	0	1	0.74
*bla*_SHV-155_	0	0	20	14.71
*bla*_SHV-156_	0	0	1	0.74
*bla*_TEM_	103	52.02	124	91.18
*bla*_TEM-1B_	102	51.52	121	88.97
*bla*_TEM-213_	1	0.51	0	0
*bla*_TEM-208_	1	0.51	3	2.21
*bla*_OXA_	0	0	19	13.97
*bla*_OXA-1_	0	0	18	13.24
*bla*_OXA-23_	0	0	1	0.74
*bla*_OXA-66_	0	0	1	0.74

The proportion of strains carrying the *bla_CTX-M_* gene was 55.05% in Eastern China and 94.85% in Central China, with the highest detection rate for *bla_CTX-M-65_* (Eastern China 46.97%, Central China 91.91%). The proportion of strains carrying the blaTEM gene was 52.02% (Eastern China) and 91.18% (Central China), with *bla*_TEM-1B_ being the most prevalent (Eastern China 51.52%, Central China 88.97%). The carrying rate of the *bla*_OXA_ gene was lower, with no detection in Eastern China, while the carrying rate in Central China was 13.97%, with *bla*_OXA-1_ accounting for 13.24%. Furthermore, the study also detected genes associated with resistance to aminoglycosides, quinolones, fosfomycin, tetracyclines, macrolides, and sulfonamides, and statistical analysis of their distribution in both regions was performed (Table 3).

**Table 3:** Other relevant resistance genes carried by cKP.

Drug resistance genes	Eastern China		Central China	
	Number of positive strains	Percentage (%)	Number of positive strains	Percentage (%)
Fosfomycins				
*fosA*	66	33.33	131	96.32
*fosA3*	91	45.96	4	2.94
*fosA14*	41	20.71	1	0.74
Tetracyclines				
*tet(A)*	31	15.66	78	57.35
*tet(D)*	0	0	8	5.88
*tet(B)*	0	0	1	0.74
*tet(M)*	0	0	1	0.74
Macrolides				
*mph(A)*	7	3.54	22	16.18
*mph(E)*	1	0.51	10	7.35
*mph(A) + mph(E) + msr(E)*	0	0	2	1.47
*mph(E) + msr(E)*	2	1.01	2	1.47
Sulfonamides				
*sul1*	41	20.71	78	57.35
*sul2*	99	50.00	25	18.38
*sul13*	13	6.57	2	1.47
*dfrA1*	2	1.01	35	25.74
*dfrA14*	85	42.93	21	15.44
*dfrA12*	4	2.02	13	9.56
*dfrA27*	4	2.02	0	0
Chloramphenicol				
*catA2*	105	53.03	83	61.03
Amide alcohols				
*floR*	2	1.01	0	0
*cmlA1*	2	1.01	0	0
Rifampicin class				
*ARR-3*	4	2.02	13	9.56

### 3.3 Capsule serotype distribution and virulence gene analysis

**Figure 3:**
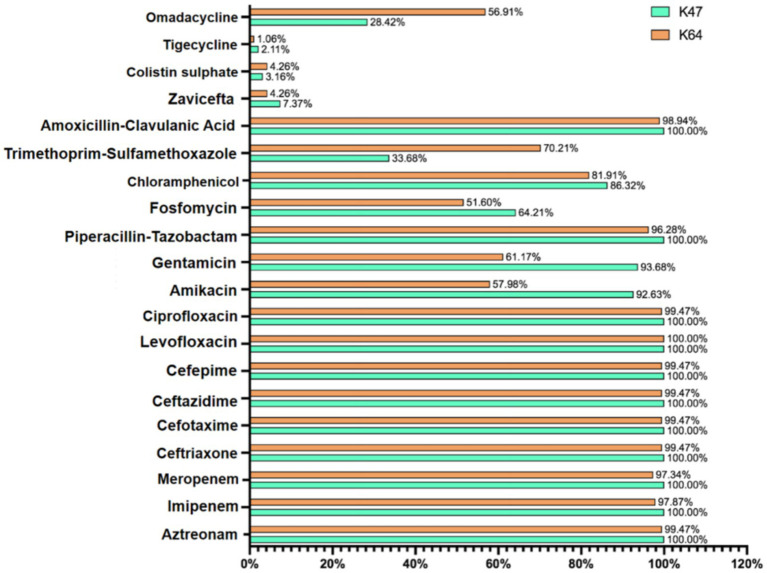
The antimicrobial resistance rates of the K47-cKP and K64-cKP strains.

Among the 334 cKP strains, 292 strains (87.4%) were identified with capsule serotypes. In Eastern China, 162 strains (81.82%) were positive for capsule serotypes. Among these, the predominant serotype was K64 (67.68%), followed by K47 (13.64%) and K61 (0.51%). In Central China, 130 strains (95.59%) were positive for capsule serotypes, with K47 (50.00%) being the predominant serotype, followed by K64 (40.44%) and others such as K25 (2.94%) (Table 4). The resistance rates to amikacin and gentamicin in K47-cKP were significantly higher than in K64-cKP strains (Table 5 and Figure 3).

**Table 4:** Capsular serotype distribution of cKP.

Capsular serotype	Eastern China		Central China	
	Number of positive strains	Percentage (%)	Number of positive strains	Percentage (%)
K64	134	67.68	55	40.44
K47	27	13.64	68	50.00
K25	0	0	4	2.94
K15	0	0	2	1.47
K10	0	0	1	0.74
K61	1	0.51	0	0

**Table 5:** Resistance rates of different drugs in capsular serotypes K47 and K64.

The name of the drug	Number of K47 resistant strains	Number of K64resistant strains
Gentamicin	95	187
Amikacin	95	184
Meropenem	95	183
Imipenem	95	187
Doripenem	95	187
Ertapenem	95	187
Meropenem-vaborbactam	95	187
Levofloxacin	95	188
Ciprofloxacin	95	187
Amikacin	88	109
Cefepime	89	115
Piperacillin-tazobactam	95	181
Cefotaxime	61	97
Ceftriaxone	82	154
Trimethoprim-sulfamethoxazole	32	132
Aztreonam-clavulanic acid	95	186
Imipenem-avibactam	7	8
Colistin	3	8
Tigecycline	2	2
Eravacycline	27	107

**Figure 4:**
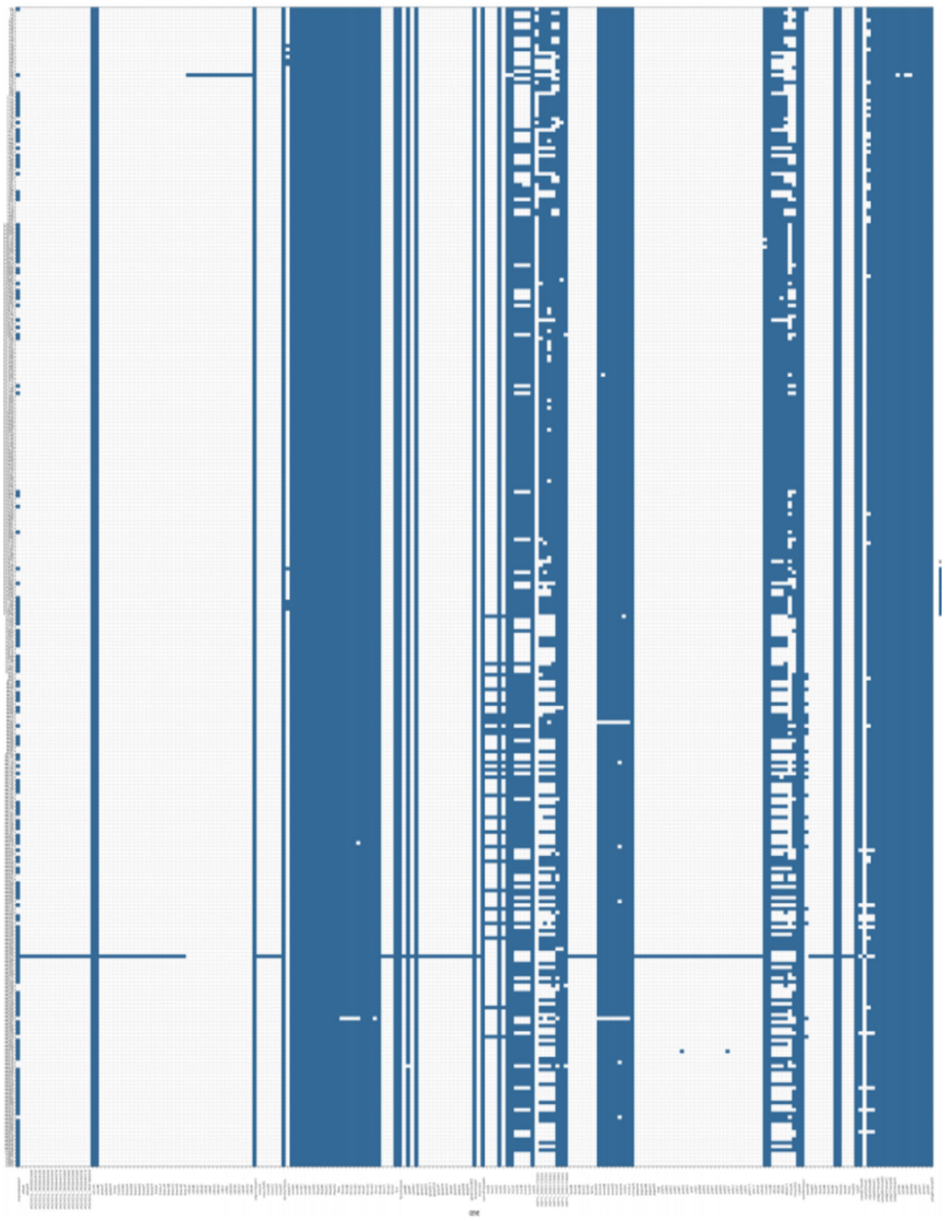
This includes all the virulence genes found in isolate 334 cKP.

Virulence factor analysis using the VFDB database showed that cKP strains carried various virulence factors, including secretion systems, adhesins, efflux pumps, iron acquisition systems, and capsule synthesis regulatory genes. In Eastern China, all cKP strains carried complete type I (fimH) and type III (mrkD) fimbrial adhesin receptor genes. In Central China, only two strains lacked some adhesin receptor genes. No strains were found to carry type IV pili-related virulence genes. In Eastern China, the CPS synthesis regulatory genes rmpA and rmpA2 were present in 40.76 and 61.41% of strains, respectively. In Central China, these percentages were 33.01 and 72.99%, respectively. The iron uptake system-related genes iucA, iroB, and the siderophore receptor protein encoding gene iutA were also identified. These five key virulence genes—rmpA, rmpA2, iucA, iutA, and iroB—are typically found on virulence plasmids, and thus are referred to as virulence plasmid-associated genes. However, no strains in this study were found to carry the iroB gene. Therefore, the high virulence gene profile of ST11-cKP strains was identified as rmpA + rmpA2 + iucA + iutA, present in 106 strains, suggesting that ST11 strains did not acquire complete virulence plasmids. All ST11-K64-cKP strains possessed rmpA2, iucA, iucB, iucC, iucD, and iutA genes, which are key genes for distinguishing ST11-K64-cKP from ST11-K47-cKP strains. The presence of these genes promotes the acquisition of highly virulent ST11-cKP strains, with detailed virulence gene statistics shown in Figure 4.

### 3.4 Plasmid replicon distribution and biofilm formation capacity

We detected 13 different types of plasmid replicons among the 334 cKP strains. Most strains contained three or more types of plasmid replicons, indicating that these strains carry multiple plasmids, increasing the potential for horizontal gene transfer. These replicons were primarily categorized into resistance plasmids and virulence plasmids, with the most common types being IncFII, ColRNAI, IncR, IncFIB, and IncHI1B. In Eastern China, the predominant replicon was IncR (93.94%), followed by IncFII (89.90%), IncHI1B (62.63%), IncFIB (55.05%), and ColRNAI (54.04%). All strains from Central China carried IncR, with over 90% of strains carrying IncFII and ColRNAI plasmid replicons (Table 6).

**Table 6:** ST11-cKP plasmid replicon type distribution.

Plasmid type	Eastern China		Central China	
	Number of positive strains	Percentage (%)	Number of positive strains	Percentage (%)
IncFII	178	89.90	134	98.53
ColRNAI	107	54.04	123	90.44
IncR	186	93.94	136	100.00
IncFIB	109	55.05	68	50.00
IncHI1B	124	62.63	45	33.09
IncN	5	2.53	24	17.65
IncI1	5	2.53	1	0.74
IncX1	1	0.51	0	0
IncX3	3	1.52	0	0
IncN3	1	0.51	0	0
IncHI2A	1	0.51	0	0
IncFIC	1	0.51	1	0.74

**Figure 5:**
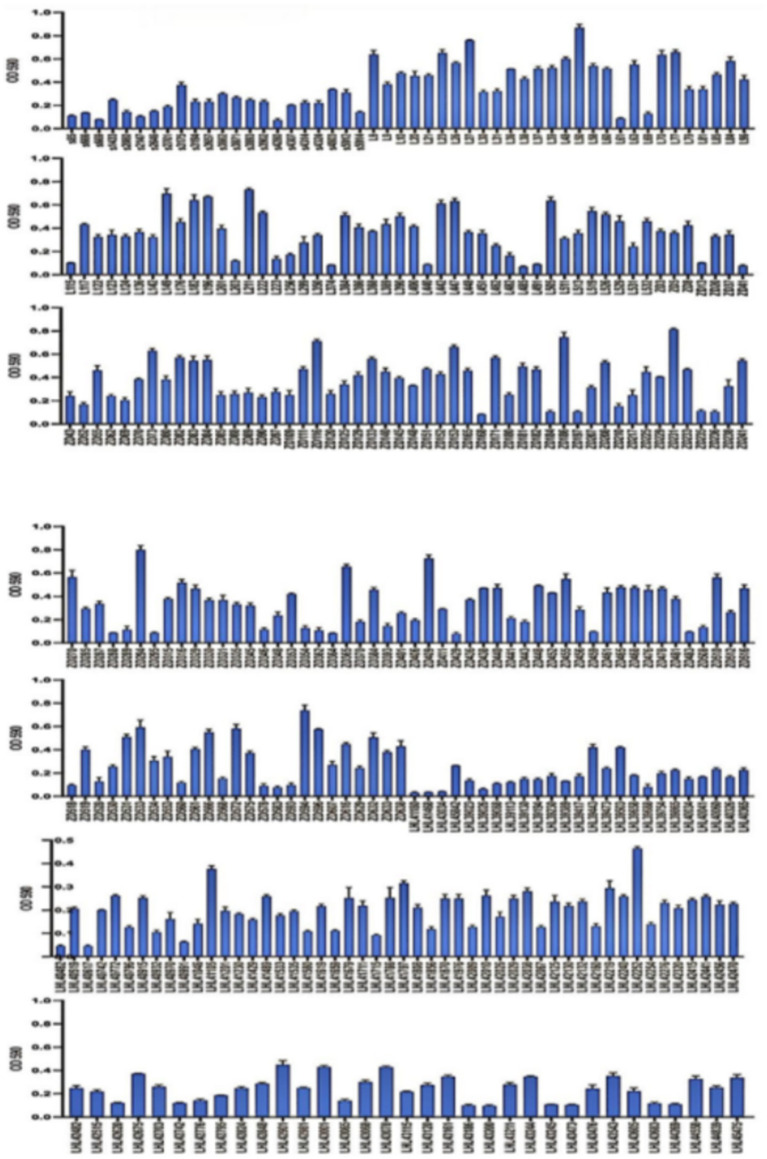
Virulence characterization of 334 carbapenem-resistant *K. pneumoniae* (cKP) strains.

**Figure 6:**
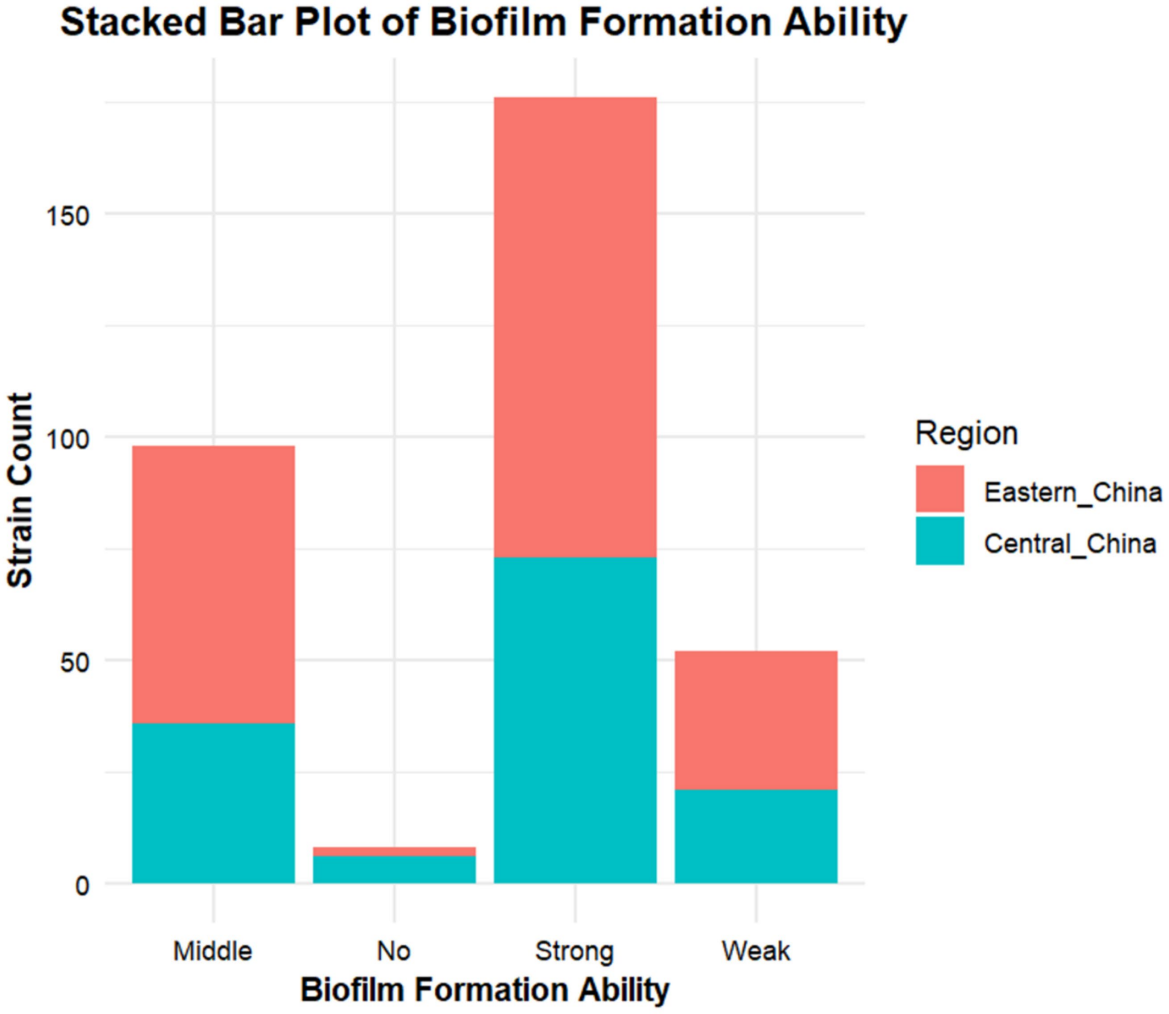
The biofilm formation ability of strains was different in different regions.

**Figure 7:**
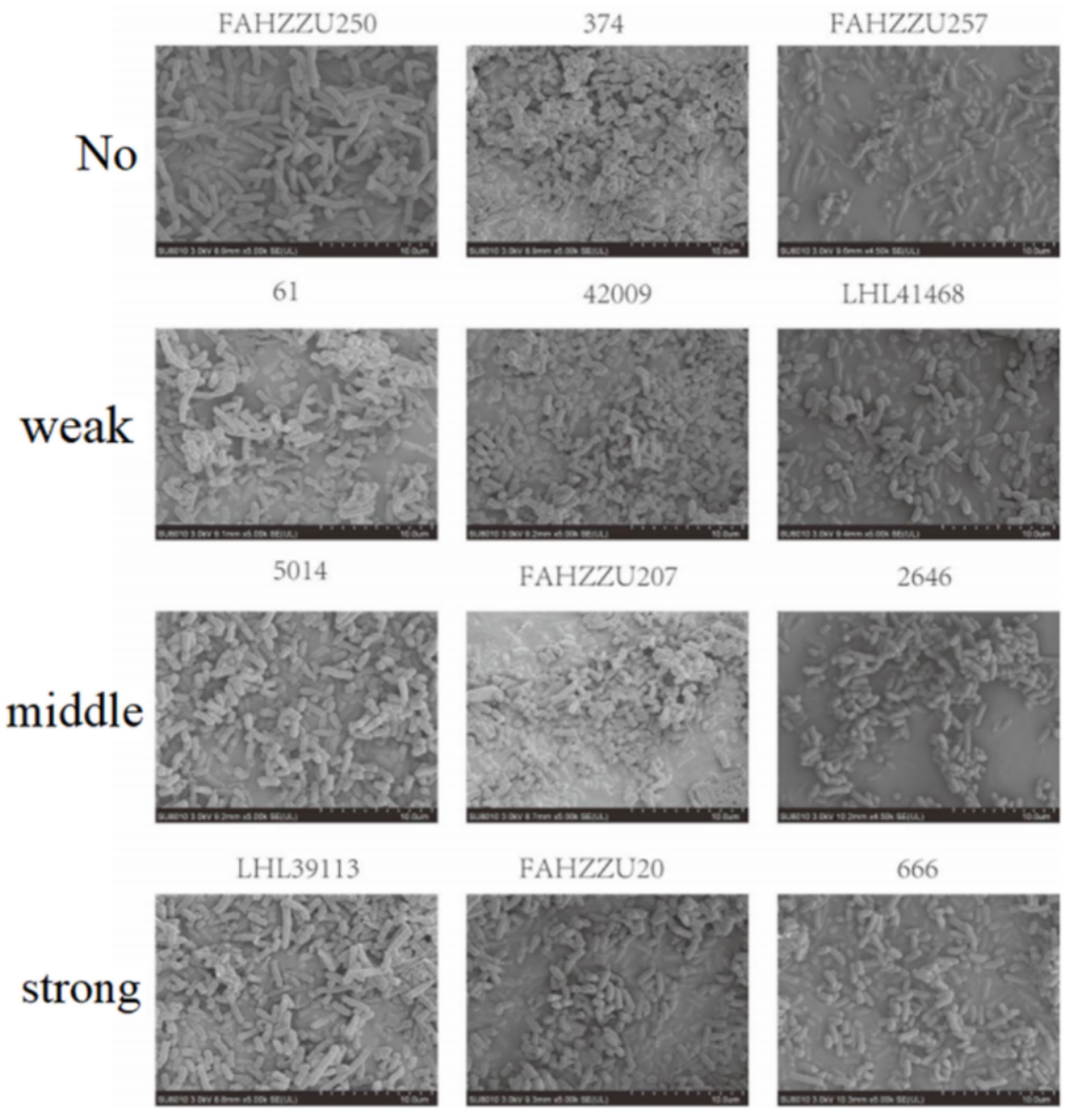
cKP forms biofilms with varying strengths that are observed using SEM.

According to crystal violet assays, of the 334 cKP strains, 8 did not form biofilms, 51 formed weak biofilms, 98 formed moderate biofilms, and 176 formed strong biofilms (Figure 5). In Eastern China, 2 strains did not form biofilms, 31 formed weak biofilms, 62 formed moderate biofilms, and 103 formed strong biofilms (Figure 6). In Central China, 6 strains did not form biofilms, 21 formed weak biofilms, 36 formed moderate biofilms, and 73 formed strong biofilms. We randomly selected 3 strains from different biofilm formation groups for scanning electron microscopy (SEM) visualization, with results shown in Figure 7.

Regional analysis revealed that in Eastern China (*n* = 198), the proportion of strong biofilm formers was 58.1% (103 strains), significantly higher than in Central China at 53.7% (73 strains), with a *p*-value < 0.05 (Table 6).

SEM results further confirmed the biofilm formation patterns, with randomly selected strains from different biofilm strength groups (strong/moderate/weak) showing biofilm structures consistent with the quantitative assay results (Figure 7).

### 3.5 Effects of Lactobacillus intervention on CRKP biofilm and metabolism

#### 3.5.1 Isolation of Lactobacillus and experimental design for CRKP intervention

We enriched 23 collected breast milk samples in MRS broth, plated them, and after anaerobic culture of individual colonies, identified them by mass spectrometry, which led to the selection of 1 strain of *Lactobacillus fermentum* and 2 strains of *Lactobacillus gasseri*.

**Figure 8:**
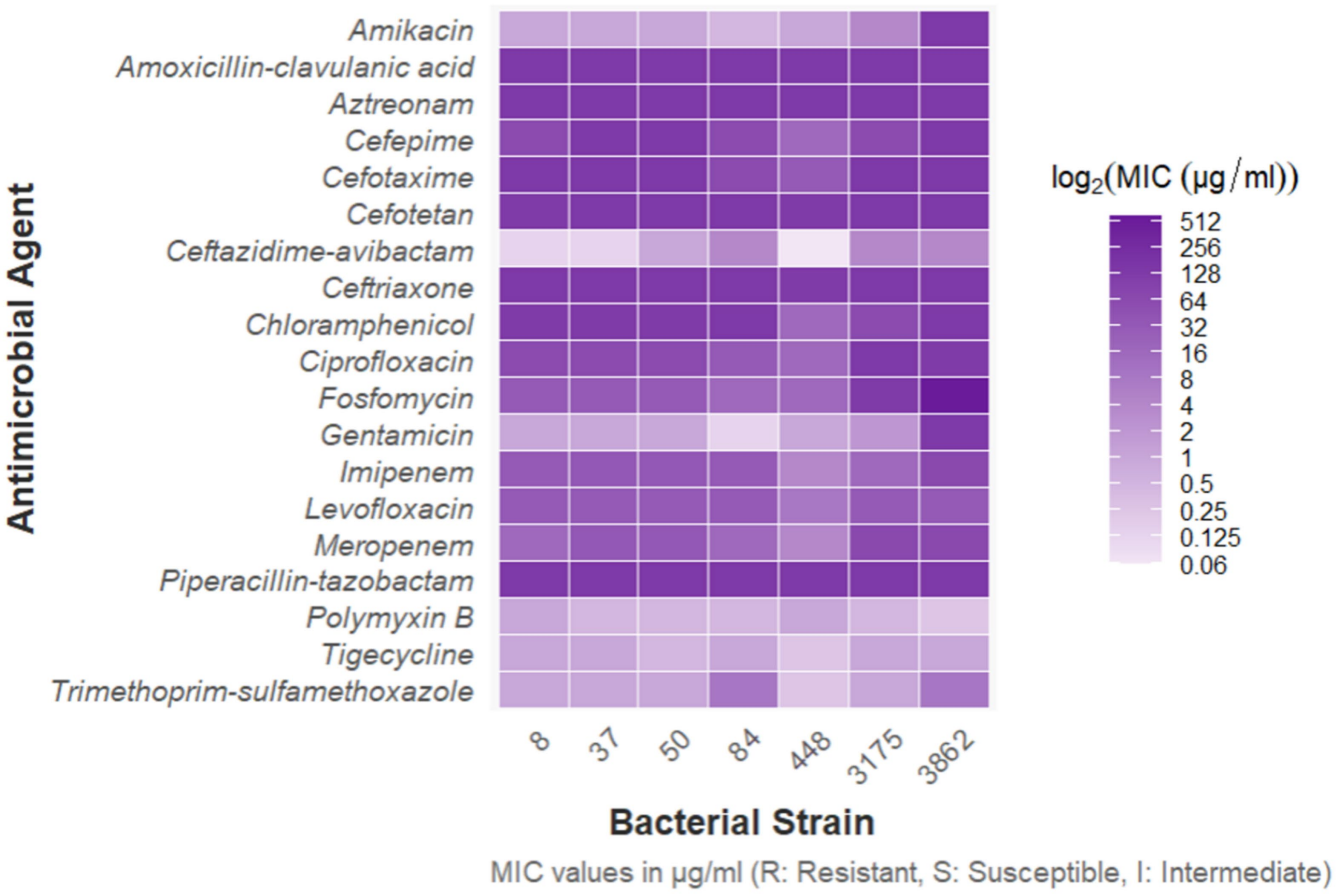
Antimicrobial susceptibility results of 7 CRKP strains.

From the group of 176 strong biofilm-forming strains in the cKP biofilm experiment, we randomly selected 7 CRKP strains for the Lactobacillus intervention experiment to inhibit biofilm formation. Among these, 3 strains were from blood specimens, and 4 strains were from fecal specimens, with the drug sensitivity test results shown in Figure 8.

#### 3.5.2 Lactobacillus’s effect on biofilm formation and scanning electron microscopy

**Figure 9:**
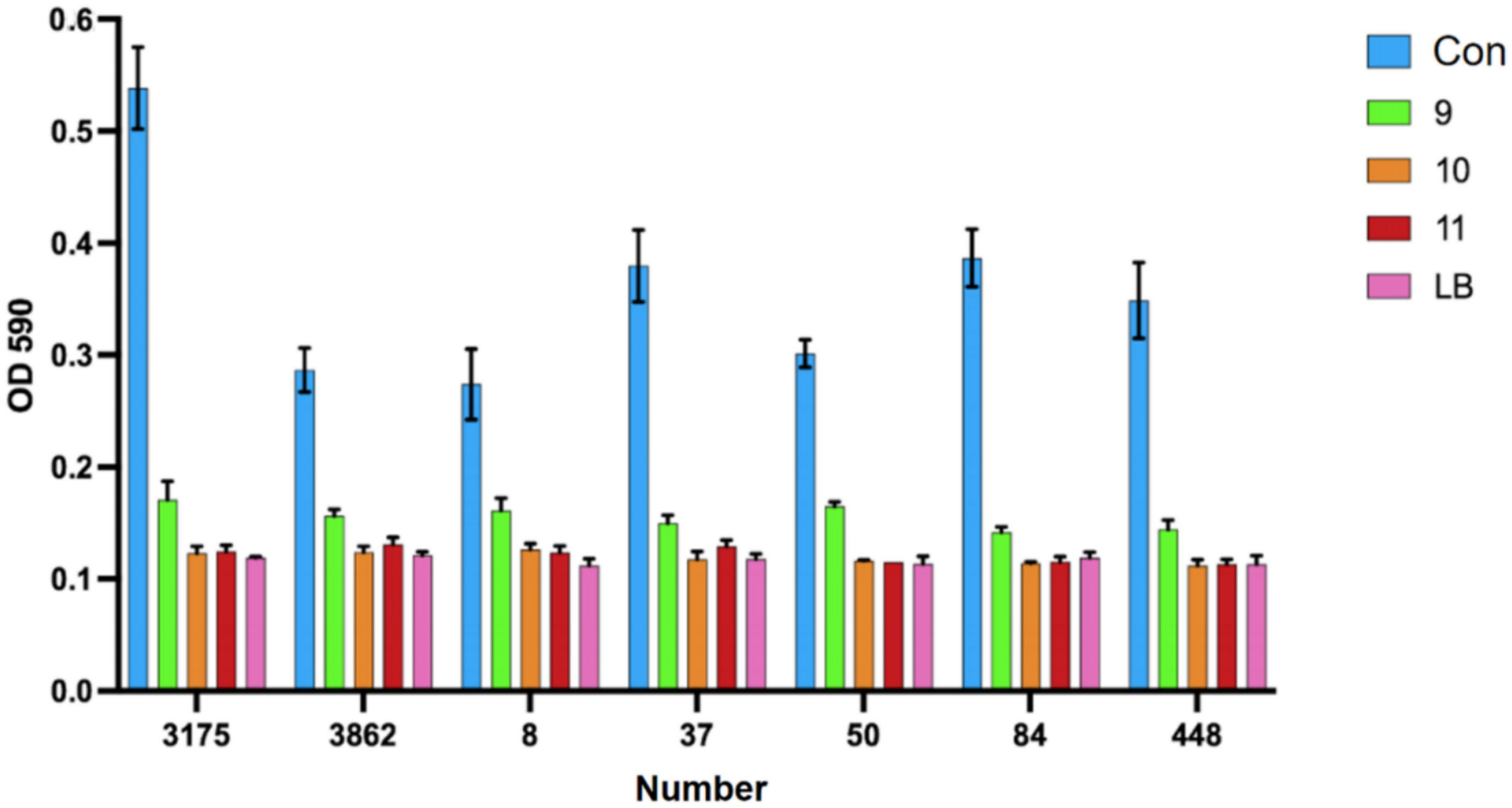
The phenomenon of inhibition of biofilm formation of Carbapenem-resistant *Klebsiella pneumoniae* by various strains of Lactobacillus.

We used the crystal violet staining method to see how *Lactobacillus fermentum* and *Lactobacillus gasseri* affected CRKP biofilm formation. After co-cultivation, the ability to form biofilm decreased (Figure 9). Among them, Group 9 was the *Lactobacillus fermentum* treatment group, and Groups 10 and 11 were the *Lactobacillus gasseri* treatment groups. To further look at the effects of Lactobacillus on CRKP cell morphology, we used scanning electron microscopy to observe the structural changes of CRKP cells treated and untreated with Lactobacillus.

**Figure 10:**
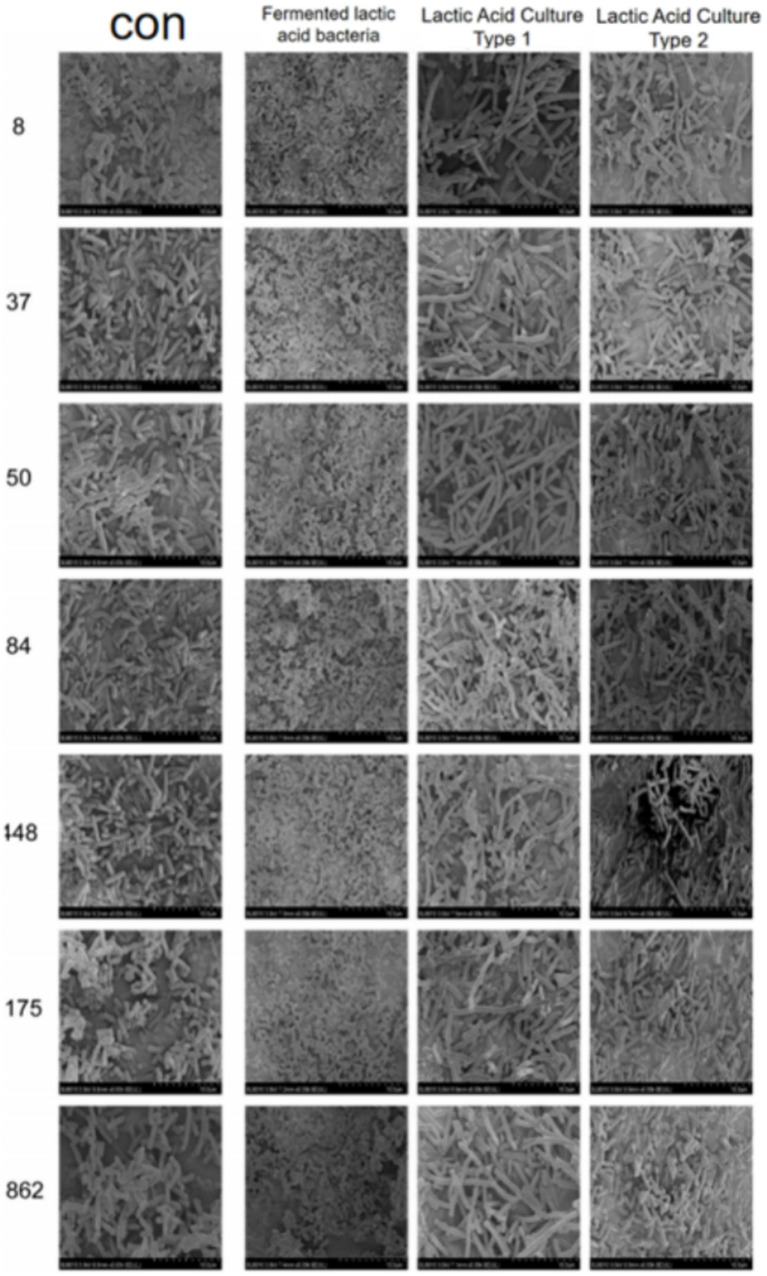
Images from a scanning electron microscope of CRKP from different Lactobacillus treatment groups.

As shown in Figure 10, the CRKP in the control group appeared rod-shaped with blunt ends and faintly visible pili. After treatment with *Lactobacillus fermentum*, CRKP changed to a short rod shape; whereas after treatment with *Lactobacillus gasseri*, CRKP appeared as long rods with a smoother surface and exhibited cracks and channels.

#### 3.5.3 Analysis of metabolomics results

**Figure 11:**
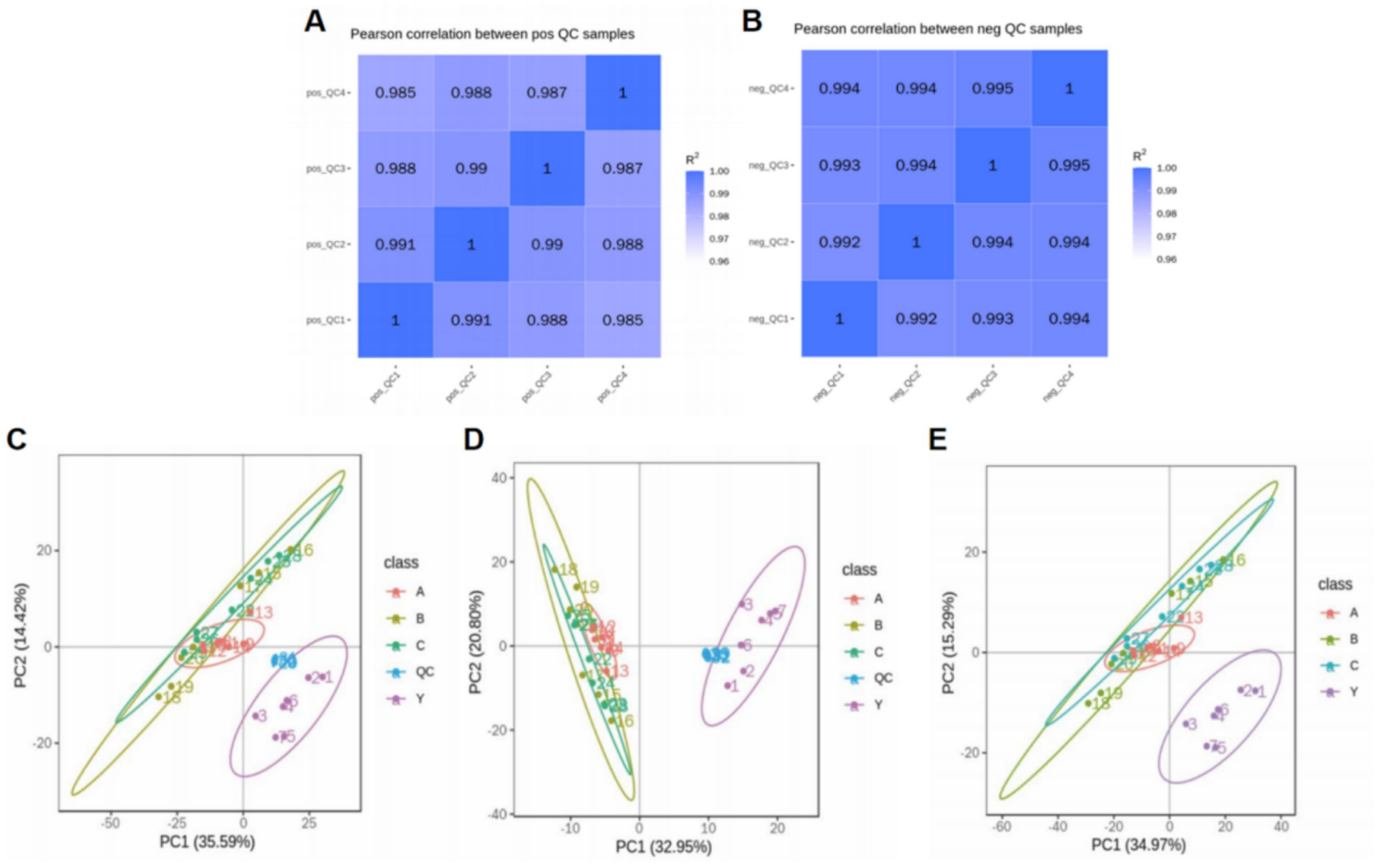
Analysis of metabolomics results. **(A,B)** Relevance of QC samples. **(C–E)** Overall PCA plot of all metabolic sample data.

By calculating the Pearson correlation coefficients between QC samples and based on the relative quantitative analysis results of metabolites, it was shown that the correlation among QC samples was high (*R*^2^ close to 1), indicating that the experimental detection process was stable, and the data quality held up well. The correlation coefficients of all QC samples exceeded 0.98, further proving good consistency among samples and high credibility of the experimental data. Although there may be some errors in the metabolite extraction and analysis process, the small differences among QC samples show that the experimental method was pretty stable and ensured data quality. PCA analysis showed that the metabolomics samples of CRKP treated with *Lactobacillus fermentum* and both *Lactobacillus gasseri* strains were more clustered together in Figure 11, further confirming the excellent data quality of this experiment, stable experimental methods, and high system reliability.

**Figure 12:**
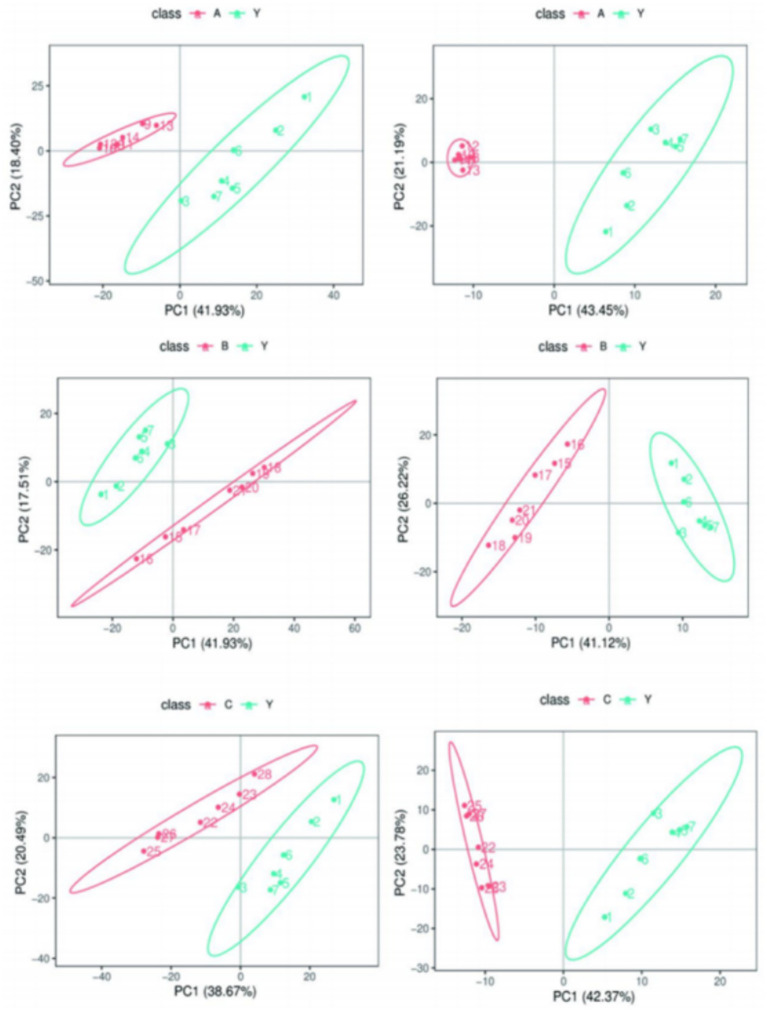
The overall distribution trend among the two sample groups.

Metabolomics research involves complex multivariate datasets, making it necessary to use multivariate analysis techniques for data processing and analysis. PCA analysis results showed that the metabolomics samples of CRKP treated with *Lactobacillus fermentum*, *Lactobacillus gasseri* 1, and *Lactobacillus gasseri* 2 were more concentrated in distribution, indicating good internal consistency within the experimental groups. At the same time, the significant distribution differences between the experimental and control groups suggest that Lactobacillus treatment can significantly alter the metabolic state of CRKP (Figure 12).

**Figure 13:**
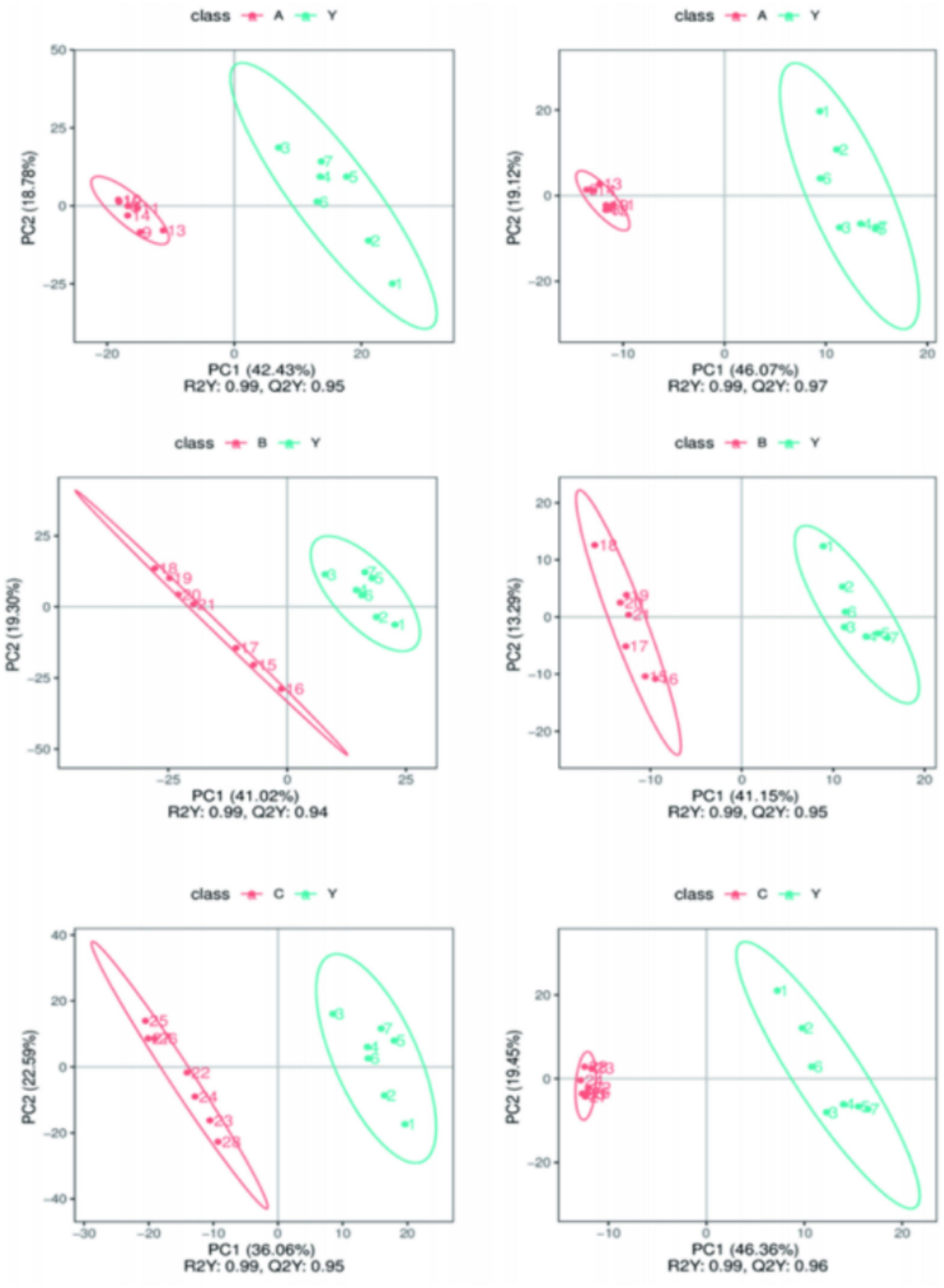
Comparing samples from two groups in Partial Least Squares Discriminant Analysis (PLS-DA) results.

PLS-DA analysis further verified the impact of Lactobacillus treatment on the metabolic profile of CRKP. The results of the PLS-DA model indicated that the metabolic profiles of the bacteria under different treatment conditions showed distinct grouping in spatial distribution, with the treatment groups clustering in different areas from the control group (Figure 13). This suggests that the PLS-DA model based on LC–MS/MS technology can effectively distinguish the metabolic states of bacteria under different experimental conditions.

Based on the standards of VIP value, FC value, and *p*-value (VIP > 1.0, FC > 1.5 or FC < 0.667, *p* < 0.05), differential metabolites were screened. In the metabolomics analysis of 28 samples, a total of 735 metabolites were identified in positive ion mode and 341 metabolites in negative ion mode. In the comparisons of A.vs.Y, B.vs.Y, and C.vs.Y, significantly different metabolites were screened (Figure 8).

**Figure 14:**
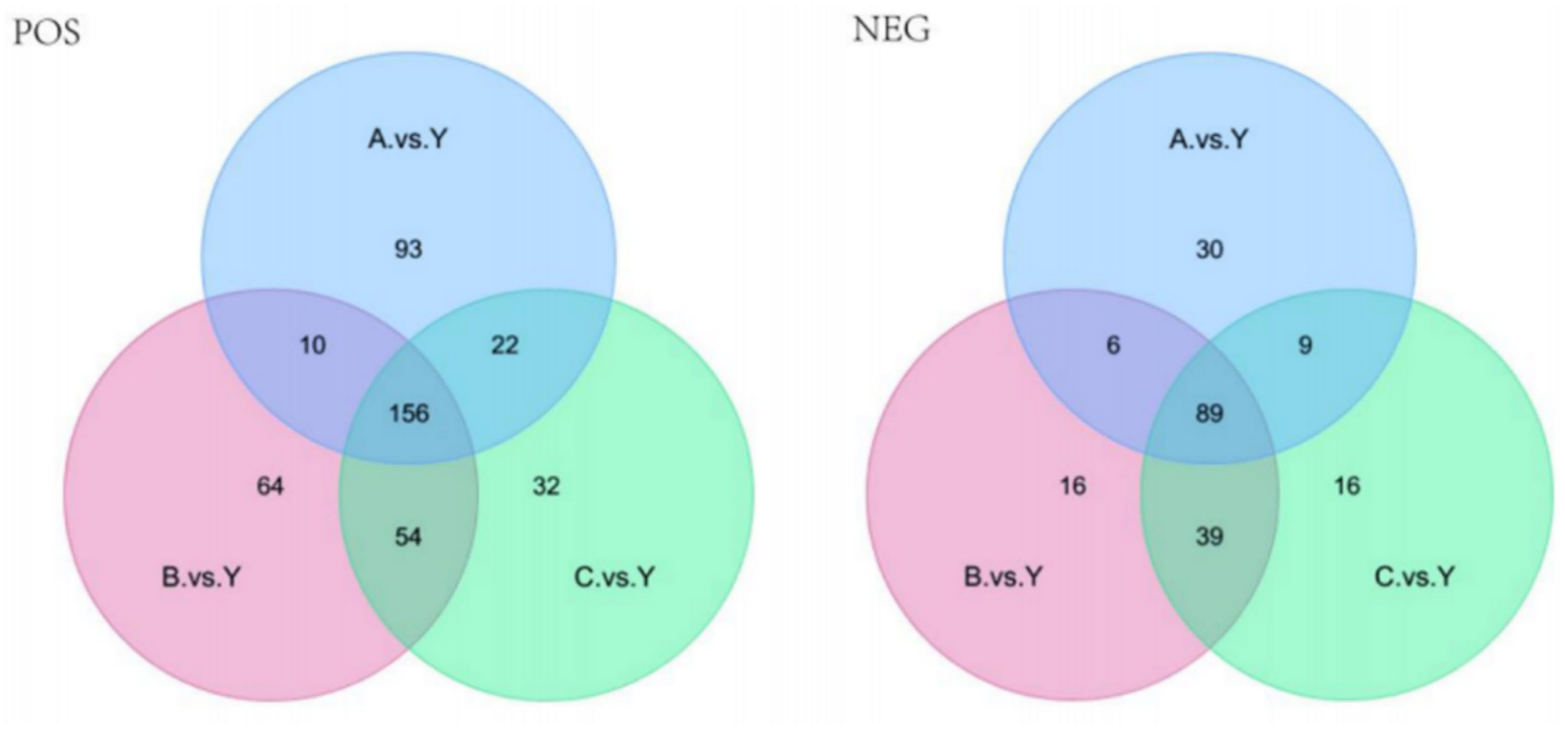
Venn diagram of differential metabolites.

In positive ion mode, there were 156 significantly different metabolites common to the 3 groups, with 93 unique metabolites in the A.vs.Y comparison group, 64 unique metabolites in the B.vs.Y comparison group, and 31 unique metabolites in the C.vs.Y comparison group; in negative ion mode, there were 89 significantly different metabolites common to the 3 groups, with 30 unique metabolites in the A.vs.Y comparison group and 16 unique metabolites in both the B.vs.Y and C.vs.Y comparison groups, as shown in Figure 14 (Table 7).

**Figure 15:**
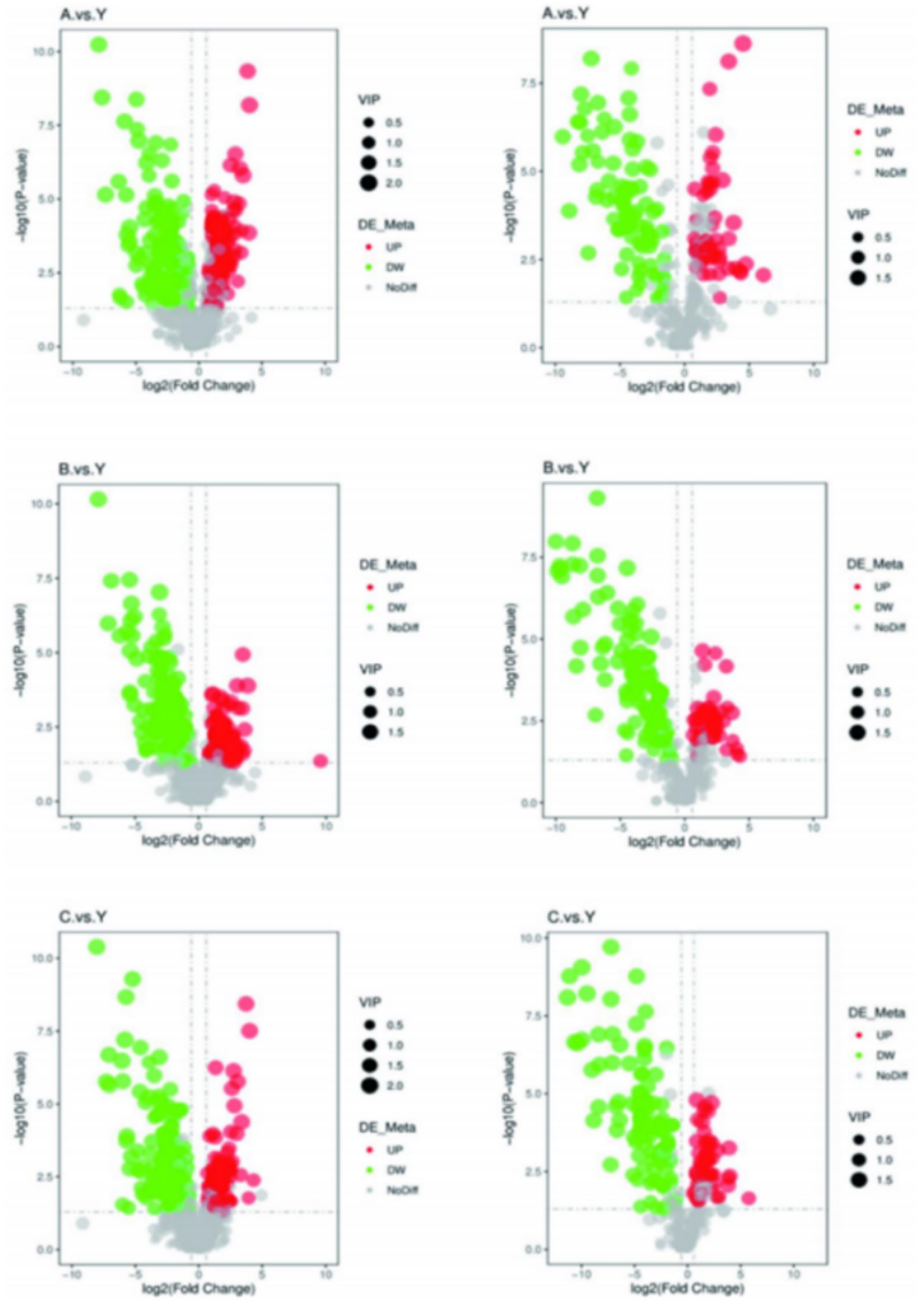
Comparing samples from two groups for the volcano plot results.

The volcano plot visually displays the changes in metabolites (Figure 15). Red dots represent significantly increased metabolites, green dots represent significantly decreased metabolites, and the size of the dots reflects the trend of VIP values.

**Table 7:** Metabolite differential screening results.

Compare samples	Total metabolite identification	Total number of metabolites that vary significantly	Significantly upregulated total metabolic totals	Significantly downregulated total metabolic total
A.vs.Y_pos	735	281	141	140
B.vs.Y_pos	735	284	119	165
C.vs.Y_pos	735	264	107	157
A.vs.Y_neg	341	134	52	82
B.vs.Y_neg	341	150	43	107
C.vs.Y_neg	341	153	52	101

#### 3.5.4 KEGG enrichment analysis

We used KEGG database analysis to find out how the different metabolites fit into metabolic pathways. Enrichment analysis of KEGG pathways through hypergeometric testing revealed the key roles of metabolites in biochemical metabolism and signal transduction.

The experimental results showed that CRKP treated with the supernatant of *Lactobacillus fermentum* showed significant enrichment in purine metabolism, cysteine and methionine metabolism, and aminoacyl-tRNA biosynthesis pathways (Figures 13A,B). Among them, the characteristic metabolites of the purine metabolism pathway included adenosine diphosphate ribose and inosine; the cysteine and methionine metabolism pathway enriched core sulfur metabolism molecules such as S-adenosyl-L-methionine and glutathione; the aminoacyl-tRNA biosynthesis pathway specifically enriched essential amino acids such as tyrosine and methionine.

**Figure 16:**
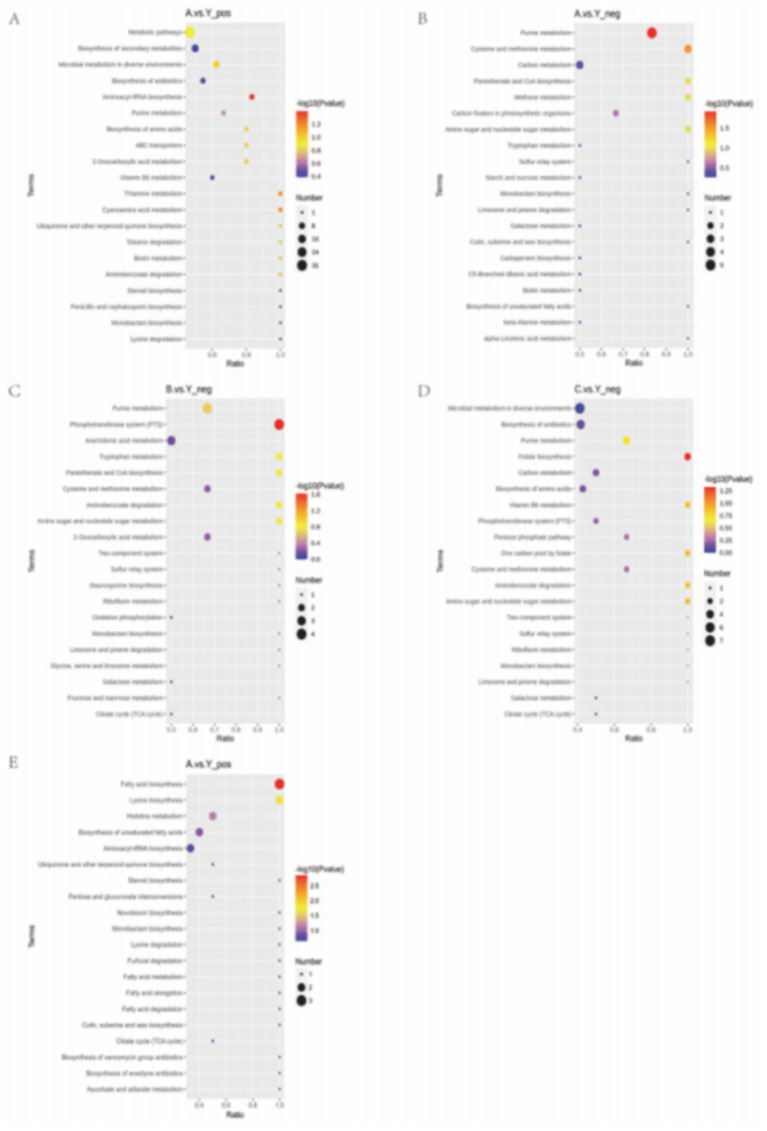
KEGG pathway enrichment analysis bubble plot.

Further analysis of the metabolomics data from the *Lactobacillus gasseri* treatment group revealed specific activation of the phosphotransferase system and folate biosynthesis pathway (Figures 13C,D). The former involves phosphorylated metabolites such as d-amino glucose, while the latter is characterized by folate and its precursor 7,8-dihydrofolate. Notably, the specific metabolites of *Lactobacillus fermentum* formed significant enrichment clusters in the fatty acid biosynthesis and lysine biosynthesis pathways (Figure 16), where long-chain fatty acids such as palmitic acid and intermediates of lysine synthesis such as *α*-ketoglutarate constituted characteristic metabolic profiles.

This study reveals through multi-level metabolic pathway analysis that Lactobacillus mainly reconstructs the metabolic homeostasis of pathogenic bacteria by regulating the nucleotide metabolism (purine metabolism), amino acid metabolism (aminoacyl-tRNA biosynthesis), and lipid metabolism (fatty acid synthesis) networks. This cross-pathway metabolic reprogramming phenomenon may be closely related to its intervention in bacterial epigenetic regulation and energy metabolism through key metabolic node molecules (such as SAM, α-KG).

## 4 Discussion

In recent years, the isolation rate of KP in China has risen sharply, becoming a significant public health concern. According to the CHINET report, KP ranks as the second most frequently isolated pathogen among all clinical isolates, with its isolation rate steadily increasing year by year. In China, the ST11 clone is the predominant type responsible for hospital outbreaks (Wang et al., 2018). In this study, 99.7% of the 334 ST11 KP strains carried KPC-type carbapenemases, while strains harboring NDM genes accounted for only 1.5%. These findings are consistent with studies on carbapenem-resistant Enterobacteriaceae (CRE) (Han et al., 2020; Iovleva and Doi, 2017; Jean et al., 2022; Suay-García and Pérez-Gracia, 2019). Among the KPC variants, only KPC-2 was identified, whereas both NDM-1 and NDM-5 subtypes were detected in NDM-positive strains. Our results indicate that most patient samples were derived from the respiratory tract, which is closely related to the colonization of KP in the respiratory tract and the ease of collecting respiratory samples. The majority of strains were isolated from the ICU, consistent with multiple studies reporting that KP infections are more common among critically ill patients (Hu et al., 2020; Qin et al., 2020; Yan et al., 2025). Contributing factors include prolonged hospital stays, severe illness, immunocompromised status, high-dose antibiotic usage, and frequent invasive procedures. These factors increase the risk of bacterial invasion and colonization. Not only in Eastern and Central China, but ICU wards in other regions have also experienced concentrated outbreaks of KPC-producing KP strains, highlighting that ICUs are a key risk factor for the spread of these resistant genes. In conclusion, all hospital departments, especially ICUs, should implement timely measures to detect CRKP carriers early, enhance disinfection protocols for wards, medical staff, and medical devices, and strengthen antibiotic stewardship. Moreover, ST11-type CRKP has been isolated in multiple hospital departments such as neurosurgery, respiratory, emergency, and general surgery departments, indicating its widespread presence in hospital settings (Konar et al., 2020).

In this study, over 95% of the 334 cKP strains were resistant to carbapenems, cephalosporins, and quinolones. Notably, all strains were 100% resistant to levofloxacin, and nearly all strains were resistant to aztreonam, imipenem, meropenem, ceftriaxone, cefotaxime, ceftazidime, ciprofloxacin, piperacillin-tazobactam, and amoxicillin-clavulanic acid. This extensive resistance to *β*-lactams is closely associated with the multiple β-lactam resistance genes carried by these strains. All ST11-cKP strains in this study carried both carbapenemase and ESBL genes, with most strains harboring two or more ESBL genes, including *bla_TEM_*, *bla_SHV_*, and *bla_CTX-M_*. Although the *bla_CTX-M_* gene is predominant in China, *bla_SHV_* was the most frequently identified gene among ST11-cKP strains in this study, followed by *bla_CTX-M_*, with the highest proportion of *bla_CTX-M-65_*, consistent with the major circulating subtype in China. Infections caused by CKP are commonly treated with aminoglycosides and fosfomycin. The susceptibility testing results in this study showed high resistance rates to ciprofloxacin (99.7%), gentamicin (71.9%), and amikacin (68.9%). Significant regional variations were observed in the resistance rates for gentamicin and amikacin between Eastern and Central China, with resistance rates of 50.51 and 54.04% in Eastern China, compared to 95.59 and 97.79% in Central China, respectively. Resistance gene analysis showed that strains carried multiple types of aminoglycoside resistance genes, with an overall carrying rate of 61.38%. In addition, a significant proportion of these strains carried genes associated with quinolone resistance, while the prevalence of resistance genes related to fosfomycin, chloramphenicol, tetracyclines, and sulfonamides was relatively low and not strongly correlated with antibiotic resistance (Mah et al., 2003).

The presence of multiple resistance genes in cKP strains severely limits the effectiveness of conventional treatment regimens. According to CLSI and EUCAST standards, only a few drugs, such as polymyxin B, tigecycline, and ceftazidime-avibactam, showed relatively high susceptibility. However, the efficacy of tigecycline in treating bloodstream infections remains controversial, with some studies suggesting it may increase mortality. Additionally, pathogens carrying KPC-2 are generally only susceptible to ceftazidime-avibactam among β-lactam antibiotics, which significantly limits available treatment options and contributes to high mortality rates. In this study, the resistance rate to ceftazidime-avibactam in Central China reached 11.76%, significantly higher than the 2.53% observed in Eastern China, possibly due to mutations in the *bla_KPC-2_* gene. The resistance rate to omadacycline in this study was as high as 48.5%, surpassing that of tigecycline, indicating that cKP strains may develop resistance to omadacycline through different mechanisms. Further analysis revealed that these omadacycline-resistant strains retained good sensitivity to polymyxin B and ceftazidime-avibactam, providing alternative treatment options for clinical management of omadacycline-resistant strains. Further research found that the omadacycline-resistant strains were predominantly ST11 and ST15, which are the dominant types in the transmission of cKP in China, particularly in hospital infections and ICUs. The major carbapenemases are KPC-2 and KPC-3, which dominate the KPC-producing KP strains in China. Clinical decision-making regarding antibiotic therapy should take into account the MLST type or carbapenemase type of the strain to facilitate more rational drug selection and improve treatment outcomes. Therefore, the characteristic studies on ST11-cKP are crucial for controlling the spread of this pathogen, promoting more accurate diagnosis, and improving treatment success rates.

The pathogenicity of cKP is primarily related to proteins encoded by virulence genes that are involved in invasion, colonization, adhesion, and phagocytosis resistance. The main virulence factors of KP include capsule polysaccharide, lipopolysaccharide, adhesins, and siderophores. All 334 cKP strains in this study carried genes encoding type I (*fimH*) and type III (*mrkD*) fimbrial adhesin receptors, indicating the widespread presence of type I and III pili in KP strains. The *fimH* gene, a virulence factor associated with type I pili, encodes FimH adhesin, an important mediator for KP adhesion to target cells. It has been reported that 90% of KP strains express type I pili, playing a critical role in immune evasion and biofilm formation. *rmpA* and *rmpA2* are important virulence factors that regulate capsule polysaccharide synthesis and are located on high-pathogenicity virulence plasmids, promoting the synthesis of capsule polysaccharides by regulating *cps* transcription (Miethke and Marahiel, 2007). Iron is an essential element for bacterial growth and reproduction. In the human body, the availability of free iron is extremely limited due to the insolubility of Fe^3+^ and the host’s restriction on Fe^2+^ and Fe^3+^ (Bailey et al., 2016). The virulence factors carried in this study are primarily related to capsule polysaccharides, such as *rmpA* and *rmpA2*, and siderophores, such as *iucA* and *iutA*. These genes exhibited a high detection rate in ST11-type CRKP, with a combined *rmpA*, *rmpA2*, *iucA*, and *iutA* virulence gene profile being present in 33.02% of ST11-type CRKP strains, classifying them as CR-hvKP. In this study, ST11-type cKP exhibited a high detection rate of virulence genes, and a considerable proportion of strains carried multiple virulence genes. There has been relatively little research on the correlation between virulence gene carriage and antibiotic resistance in ST11-type cKP. This study analyzed the correlation between virulence genes and antibiotic resistance in ST11-type cKP isolates, showing no significant correlation between the number of virulence genes and resistance patterns. This finding contrasts with the negative correlation between virulence gene expression and resistance incidence reported by Wu et al., which may be due to differences in infection pathways and biological characteristics of the cKP isolates. The presence of multiple virulence genes enhances the survival, pathogenicity, and immune evasion abilities of the bacteria, indirectly influencing their resistance. In cKP strains, plasmids can carry both virulence and resistance genes, though further research is required to confirm the interactions between multidrug resistance and virulence factors, as well as the plasmid replicons that harbor these genes. In-depth studies may uncover key molecular targets for regulating both virulence and resistance in KP, leading to the development of new antibiotics and therapeutic strategies.

Capsule polysaccharides are one of the main factors influencing the pathogenicity of cKP. Studies have shown that KP has 78 types of capsule polysaccharide K antigen serotypes (Domenico et al., 1994). In this study, 6 K antigen types were detected in the 334 cKP strains, namely K64, K47, K25, K15, K10, and K61. K64 and K47 types were predominant in both Eastern and Central China. The susceptibility results showed that K47-type strains exhibited higher resistance to all carbapenem and aminoglycoside antibiotics. In recent years, the K64-type of ST11 cKP has been increasing globally, replacing K47 as the major serotype, potentially due to the stronger virulence and infectivity of ST11-K64, which is better able to survive in the environment (Chen et al., 2023; Zhou et al., 2020; Zhou Q. et al., 2023).

Bacterial mobile elements are key factors influencing genetic diversity and evolution. Plasmids, insertion sequences, prophages, integrative conjugative elements, and other mobile elements promote the transfer of functional genes between bacterial individuals and populations. This gene flow not only helps bacteria grow and proliferate but also significantly enhances their adaptability to environmental pressures (Zhang et al., 2018). As common mobile elements, plasmids can carry multiple resistance genes and transfer them horizontally between bacteria, leading to antibiotic resistance. This study found that ST11-cKP strains carry multiple plasmid replicons, with IncR plasmids being the most prevalent in both Eastern and Central China, followed by IncFII, ColRNAI, IncFIB, and others.

Biofilm formation is one of the key factors that lead to KP colonization and persistent infections. Biofilms are complex structures formed by bacteria and their secreted extracellular polymers, such as polysaccharides and proteins (Katongole et al., 2020; Karigoudar et al., 2019; Raya et al., 2019). The formation of biofilms provides a protective barrier for bacteria, shielding them from immune cell attacks and antibodies, and enhancing their tolerance to antibiotics. The three-dimensional structure of biofilms blocks the penetration of therapeutic agents, increasing the resistance of bacteria and complicating clinical infection treatment. In this study, 326 of the 334 cKP strains (97.6%) were biofilm-forming positive, indicating a high biofilm formation ability of cKP strains.

Metabolic activity is essential for cellular life processes, and metabolomics provides a direct reflection of the cell’s physiological state. In this study, metabolomics was used to identify metabolites involved in biofilm formation after *Lactobacillus fermentum* and two *Lactobacillus gasseri* supernatants intervened in CRKP strains. We identified 735 metabolites in the positive ion mode and 341 metabolites in the negative ion mode. After comparing the three experimental groups, we found that amino acid metabolism, nucleotide metabolism, and energy metabolism were closely related to the inhibition of biofilm formation by *Lactobacillus* and their mechanisms, as these metabolic pathways are crucial for maintaining cellular structure and function.

Amino acid metabolism plays a key role in cellular responses to environmental stress. Changes in amino acid levels reflect the cell’s adaptation and response to environmental changes. This study found that *Lactobacillus* induced alterations in the intracellular levels of various amino acids in CRKP, including the downregulation of phenylalanine, tyrosine, tryptophan, cysteine, and methionine, which can generate one-carbon units and participate in amino acid conversions. This downregulation may disrupt the amino acid metabolic balance in CRKP, inhibiting biofilm growth.

Nucleotides are essential for genetic information transmission, protein synthesis, and other vital cellular processes. Our metabolomics results indicated that decreased folate levels led to reduced intermediate products of purine metabolism, such as hypoxanthine, guanosine, and adenosine, which are critical components of DNA. We hypothesize that *Lactobacillus* inhibits DNA synthesis by suppressing deoxyribonucleotide synthesis, thus affecting biofilm formation.

By combining metabolomics and morphological analysis, this study reveals that *Lactobacillus* regulates key metabolic pathways in CRKP, including nucleotide metabolism, amino acid metabolism, and energy metabolism, to effectively inhibit biofilm formation and disrupt bacterial structural integrity. However, further research is needed to explore the direct mechanisms of *Lactobacillus* intervention in CRKP infections. Future studies could integrate CRISPR-Cas9-mediated gene knockout or mouse infection models to further validate the role of *Lactobacillus* metabolites in inhibiting CRKP infections.

## Data Availability

The datasets presented in this study can be found in online repositories. The names of the repository/repositories and accession number(s) can be found in the article/supplementary material.

## Footnotes

^1^ http://www.genomicepidemiology.org

^2^ http://www.mgc.ac.cn/VFs/
